# LC-MS/MS Based Metabolomics Reveal Candidate Biomarkers and Metabolic Changes in Different Buffalo Species

**DOI:** 10.3390/ani11020560

**Published:** 2021-02-20

**Authors:** Wen Shi, Xiang Yuan, Kuiqing Cui, Hui Li, Penghui Fu, Saif-Ur Rehman, Deshun Shi, Qingyou Liu, Zhipeng Li

**Affiliations:** State Key Laboratory for Conservation and Utilization of Subtropical Agro-Bioresources, Guangxi University, Nanning 530005, China; 18377176750@163.com (W.S.); cauyx2014@163.com (X.Y.); kqcui@gxu.edu.cn (K.C.); lihui3876@163.com (H.L.); fupenghui1975@163.com (P.F.); saif_ali28@yahoo.com (S.-U.R.); ardsshi@gxu.edu.cn (D.S.)

**Keywords:** metabolomics, buffalo, milk, biomarkers, fatty metabolism

## Abstract

**Simple Summary:**

Milk and dairy products have become the most common and an essential nutrition in human life, while dairy products with high nutritional value are attracting more and more attention. Buffalo milk contains higher protein, fat, lactose, and total solid contents and has been considered as the source of superior dairy products, such as mozzarella cheese, butter, ice cream, and yoghurt. Moreover, buffalo milk contains more unsaturated fatty acids (UFAs) which are important for human health owing to their desirable physiological effects. However, the composition of milk seems to vary among different buffalo species and inadequate information is available about the underlying mechanism. Therefore, exploring the biomarkers of superior buffalo milk is crucial for the process of dairy products and buffalo breeding. In the present study, diverse significantly different metabolites were identified among the Mediterranean, Murrah, and crossbred buffalo, and the different metabolites were mainly enriched in fat synthesis related pathways which affected the end fat content in the milk. Moreover, these specific metabolites can be used as candidate biomarkers in the identification of milk quality and molecular breeding of high milk fat buffalo.

**Abstract:**

Consumers have shown more and more interest in high-quality and healthy dairy products and buffalo milk is commercially more viable than other milks in producing superior dairy products due to its higher contents of fat, crude protein, and total solids. Metabolomics is one of the most powerful strategies in molecular mechanism research however, little study has been focused on the milk metabolites in different buffalo species. Therefore, the aim of this study was to explore the underlying molecular mechanism of the fatty synthesis and candidate biomarkers by analyzing the metabolomic profiles. Milk of three groups of buffaloes, including 10 Mediterranean, 12 Murrah, and 10 crossbred buffaloes (Murrah × local swamp buffalo), were collected and UPLC-Q-Orbitrap HRMS was used to obtain the metabolomic profiles. Results showed that milk fatty acid in Mediterranean buffalo was significantly higher than Murrah buffalo and crossbred buffalo. A total of 1837/726 metabolites was identified in both positive and negative electrospray ionization (ESI±) mode, including 19 significantly different metabolites between Mediterranean and Murrah buffalo, and 18 different metabolites between Mediterranean and crossbred buffalo. We found 11 of the different metabolites were both significantly different between Mediterranean vs. Murrah group and Mediterranean vs crossbred group, indicating that they can be used as candidate biomarkers of Mediterranean buffalo milk. Further analysis found that the different metabolites were mainly enriched in fat synthesis related pathways such as fatty acid biosynthesis, unsaturated fatty acid biosynthesis, and linoleic acid metabolism, indicating that the priority of different pathways affected the milk fat content in different buffalo species. These specific metabolites may be used as biomarkers in the identification of milk quality and molecular breeding of high milk fat buffalo.

## 1. Introduction

Milk is an important source of nutrition for infants and adults, and buffalo milk seems to be a better choice due to the higher protein, fat, lactose, and total solid contents in it [1]. Moreover, the excellent physical and technological properties of buffalo milk make it more suitable for processing of superior dairy products, such as mozzarella cheese, butter, ice cream, and yoghurt [2,3]. Buffalo is the world’s second largest dairy animal, and Mediterranean buffalo, Murrah buffalo, and Nili-Ravi form the main dairy buffalo species. Studies have found that the values for the composition (milk protein, milk fat, and total solids) of diverse buffalo milk are significantly different [2]. Meanwhile, milk protein and fat content in triple-crossbred buffalo is higher than Nili-Ravi buffalo, but lower than Murrah buffalo [4]. Milk traits among different breeds are different, and genetics is one of the main factors affecting milk quality [5].

Metabolomics is an emerging discipline developed after genomics and proteomics and is a powerful platform for studying low molecular weight metabolites (MW < 1000). Numerous rapid and high-throughput techniques, such as nuclear magnetic resonance (NMR), liquid chromatography tandem mass spectrometry (LC-MS) and gas chromatography mass spectrometry (GC-MS) have been used in metabolomic studies [6,7]. The application of metabolomics to scientific research helps researchers gets a global understanding of physiological alterations in specific organs or tissues [6]. In view of the advantages of metabolomics, it has attracted more and more research attention and has been applied in lactation research aiming to develop new nutritional, genetic, and management strategies to improve and identify milk production and quality [8,9,10]. Applying metabolomic technology, the Yang group analyzed the differences of metabolites in the milk between Holstein and other minor dairy animals and found results to show that different metabolic pathways were involved in the milk synthesis of diverse mammals [7]. The Tian group screened the differential metabolites between heat stress-free and heat stressed cows by LC-MS and ^1^H NMR, and found that those metabolites could be regarded as potential biomarkers for monitoring the heat stress of lactating dairy cows [11]. Based on the milk metabolomic data and milk production traits, the Vervoort group obtained models to estimate the energy balance of early lactation cows [12]. Metabolomics exhibited a better response to phenotypes [13], and provided extraordinary help for further study in physiology and pathology during lactation and other biochemical characteristics of milk [5,9,14]. NMR metabolomic analysis of dairy cows revealed that the milk glycerophosphocholine to phosphocholine ratio can be used as a prognostic biomarker for the risk of ketosis [15]. However, compared with other methods, LC-MS is a more sensitive technology for global metabolic profiling of complex biological samples. It can detect most of the metabolites, especially the low abundance compounds [16], which thus make it suitable for biomarker identification.

Currently, little is known about the difference of metabolome in the milk between different buffalo species. Therefore, study on the metabolites in the milk of diverse buffalo species may provide further understanding about the process of milk production. In this study, we obtained the metabolome in milk of the Mediterranean buffalo, Murrah buffalo and crossbred buffalo using UPLC-Q-Orbitrap HRMS. The differences in metabolite profiles may provide novel understanding about the molecular mechanism of milk production, and the specific metabolites in different buffalo species may be used as biomarkers in the identification of milk quality and molecular breeding.

## 2. Materials and Methods

### 2.1. Sample Collection

In this study, Buffalo milk samples were collected from purebred Mediterranean buffalo (*n* = 10), Murrah buffalo (*n* = 12) and crossbred buffalo (Murrah × local swamp buffalo, *n* = 10) in the Buffalo Breeding Farm in the Buffalo Research Institute, Chinese academy of agricultural sciences, Nanning, Guangxi, China. The Mediterranean buffalo and the Murrah buffalo are locally bred from imported purebred breeds. The crossbred buffalo in this study refers to a hybrid of Murrah buffalo and local swamp buffalo. All of the selected animals were of similar parity (2 or 3) during mid-lactation (100–200 d). All of the buffalos were maintained and reared under the same feeding and management regime in the farm. Buffalo milk samples separated into 1.5 mL centrifugal tubes were transported to the laboratory at low-temperature and stored at −80 °C until further experiment. The rest of the buffalo milk samples were collected in 50 mL centrifugal tubes and used for milk composition analysis.

### 2.2. Milk Composition Determination and Metabolite Extraction

The protein, fat, lactose, and total solids contents of milk from Mediterranean buffalo (*n* = 10), Murrah buffalo (*n* = 12) and crossbred buffalo (*n* = 10) were analyzed by a multifunction analyzer for dairy products (MilkoScan FT-120, FOSS Electric A/S, Hillerod, Denmark). Statistical analyses were performed by using Duncan’s multiple comparison with SPSS 19.0 software (IBM-SPSS Statistics, IBM Corp., Armonk, NY, USA). Variations in milk protein, fat, lactose, and total solids (TS) of researched buffalos were analyzed using one-way analysis of variance (ANOVA). Results were expressed as mean ± standard error (SEM), and differences of *p* < 0.05 were considered to be significant.

For methods of metabolite extraction from buffalo milk refer to several previous studies [11,17]. In brief, the milk samples were placed in the refrigerator at 4 °C to unfreeze. Then 50 μL of buffalo milk was mixed with 350 μL of the solvents, methanol (Sigma-Aldrich, Steinheim, Germany) and methyl-tert-butyl-ether (MTBE, Sigma-Aldrich, Steinheim, Germany) in a ratio of one to one. In this study, vitamin E acetate (Solarbio Science & Technology Co., Ltd., Beijing, China) was added as an internal standard (IS), at a final concentration of 25 ppm. The mixture was subjected to a vortex for 3 min, then stood for 10 min and finally centrifuged at 12,000 rpm for 15 min at 4 °C. The supernatant was put in a 0.22 µm filter (MilliporeSigma, St. Louis, MO, USA) and centrifuged at 12,000 rpm for 5 min at 4 °C. The collected filtrate in the centrifuge tube was stored at −80 °C until the experiment. Quality Control (QC) samples were prepared by pooling an equal volume of milk metabolite extract of each sample from Mediterranean buffalo, Murrah buffalo, and crossbred buffalo.

### 2.3. LC-MS/MS Analysis

LC-MS/MS analyses were performed by a Dionex UltiMate 3000 UHPLC (Thermo Fisher Scientific Inc., Waltham, MA, USA) system coupled with a Q Exactive mass spectrometer (Thermo Fisher Scientific Inc., Waltham, MA, USA). In on-line UPLC-MS applications, data-dependent acquisition was applied. In order to detect as many compounds as possible, both positive and negative ion modes were performed for each sample with a heated electrospray interface (HESI-Ⅱ). Each sample was injected twice, with one injection in positive ion mode and another one in negative ion mode. Every sample was injected onto a Hypersil GOLD HPLC column (50 × 2.1 mm^2^ × 1.9 μm, Thermo Fisher Scientific Inc., Massachusetts, USA) at a linear gradient at a flow rate of 0.3 mL/min and a total run time of 25 min. The mobile phase in both positive and negative ion modes was constituted as solution A (10 mM ammonium formate in water) and solution B (10 mM ammonium formate in methanol). The solvent gradient was set as follows: 0∓2 min, 5% B; 2∓5 min, 5–30% B; 5∓19 min, 30–99% B; 19−22 min, 99% B; and 22∓25 min, 5% B. 

The Q-Exactive mass spectrometer was operated in positive or negative polarity mode with spray voltage 3.5 kV and capillary temperature of 320 °C. The flow rate of sheath gas was 30 psi and aux gas was 10 arb. The full scan mode scanned from m/z 100–1000, maximum IT was 100 ms and the AGC target was 3 × 10^6^. The scanning mode of mass spectrometry was data-dependent acquisition (DDA), and the dd-MS^2^’s AGC target was 10^5^, maximum IT was 50 ms, and isolation window was 2.0 m/z.

### 2.4. Data Analysis

Compound Discoverer 3.0 (CD 3.0) software (Thermo Fisher Scientific Inc., Massachusetts, America) was applied to process and analyze the mass spectrum data, for instance, selection of spectra, peak picking and retention times alignment, molecular formula assignment, and candidate comparison with the mz-Cloud database. The *p* values were also obtained by CD 3.0 software with Student’s *t* test. The parameters of peak alignment were set as follows: retention time tolerance = 0.2 min, mass tolerance = 5 ppm. The other parameters of peak picking and peak area quantification were set as follows: mass tolerance = 5 ppm, intensity tolerance = 30%, S/N = 3 and minimum peak intensity = 100,000.

Then multivariate statistical analysis including unsupervised principal component analysis (PCA) and orthogonal partial least-squares discriminant analysis (OPLS-DA) was performed using SIMCA-P 14.1 software package (Umetrics, Umeå, Sweden). The permutation test (200 times) was performed to further validate the OPLS-DA model. The variable importance in the projection (VIP) was obtained by OPLS-DA. In this report, we define significant differential metabolites with VIP-value > 1 and *p*-value ≤ 0.05 [7,18]. Hierarchical cluster analysis (HCA) was carried out via TBtools v1.046 (TBtools: An Integrative Toolkit Developed for Interactive Analyses of Big Biological Data).

### 2.5. Metabolic Pathway Analysis

MetaboAnalyst 4.0 [19] were used for the pathway analysis based on database source including the Kyoto Encyclopedia of Genes and Genomes [20], the human metabolome database [21], the Chemical Entities of Biological Interest [22], the metabolite and tandem MS database [23] and the PubChem database [24,25].

## 3. Results

### 3.1. Routine Analysis of Milk Composition

Milk composition of Mediterranean buffalo (defined as group D, *n* = 10), Murrah buffalo (defined as group M, *n* = 12) and crossbred buffalo (Murrah × local swamp buffalo, defined as group Z, *n* = 10) were analyzed and are presented in Table 1. Results showed that the fat and total solids (TS) content in group D is significantly higher than that of the other two groups, while the protein content in group D is significantly lower than that of group Z, and of comparable level with group M. The milk composition of Murrah buffalo was similar to that of crossbred buffalo and no significant difference was found in the lactose content of the three buffalo species.

### 3.2. Milk Metabolome Profiles

**Figure 1:**
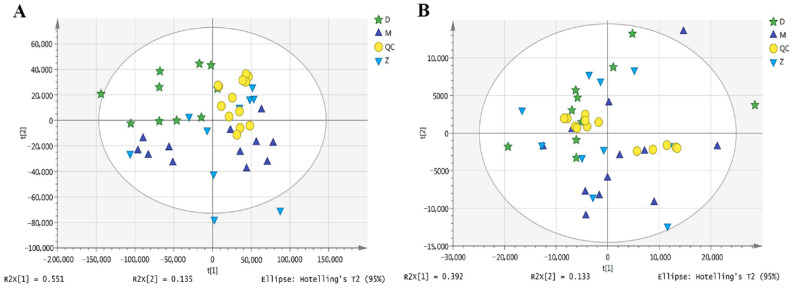
The principal component analysis (PCA) scores plot of metabolomic data in both positive (**A**) and negative (**B**) ionization modes. D indicates milk samples from Mediterranean buffalo; M indicates milk samples from Murrah buffalo; QC means samples of Quality control; Z indicates milk samples from crossbred buffalo.

**Figure 2:**
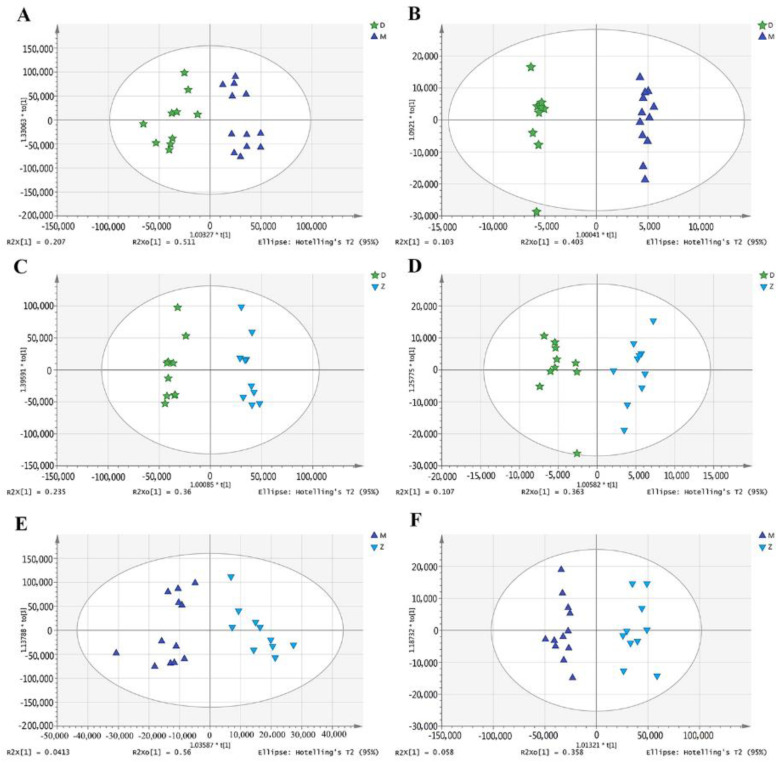
The orthogonal partial least squares discriminate analysis (OPLS-DA) of metabolomic data in both positive (**A**,**C**,**E**) and negative (**B**,**D**,**F**) ionization modes. D indicates milk samples from Mediterranean buffalo; M indicates milk samples from Murrah buffalo; Z indicates milk samples from crossbred buffalo.

Metabolites profiling of Mediterranean buffalo, Murrah buffalo, and crossbred buffalo milk was performed by UPLC-Q-Orbitrap HRMS, and 1837/726 metabolites were identified in ESI± mode, respectively (Supplementary Materials Tables S1 and S2). The representative spectra obtained in ESI± modes gave an overview of the metabolic profiles from milk samples (Supplementary Materials Figure S1). The principal component analysis (PCA) of the metabolome data showed that group D was clearly separated from other groups in positive ion mode, while no distinctive separation trend among groups was found in the negative ion mode (Figure 1). Further analysis found that the correlation of QCs in both ESI± modes, calculated by Pearson correlation coefficient, showed that both of the coefficients were above 0.9 (Supplementary Materials Figure S2), indicating the good stability, repeatability, and accuracy of the mass spectrometry data. However, the PCA of QCs showed that the separation was also only found in the ESI+ modes, demonstrating that PCA is not enough to evaluate the difference among the groups (Figure 1). Therefore, we performed the orthogonal partial least squares discriminate analysis (OPLS-DA) and distinctive separation among groups was found in both ESI± modes (Figure 2). Almost all of the samples in the score plots were within the 95% Hotelling’s T-squared ellipse, indicating that few outliers were present in samples. 

### 3.3. Identification of Differential Metabolites

**Figure 3:**
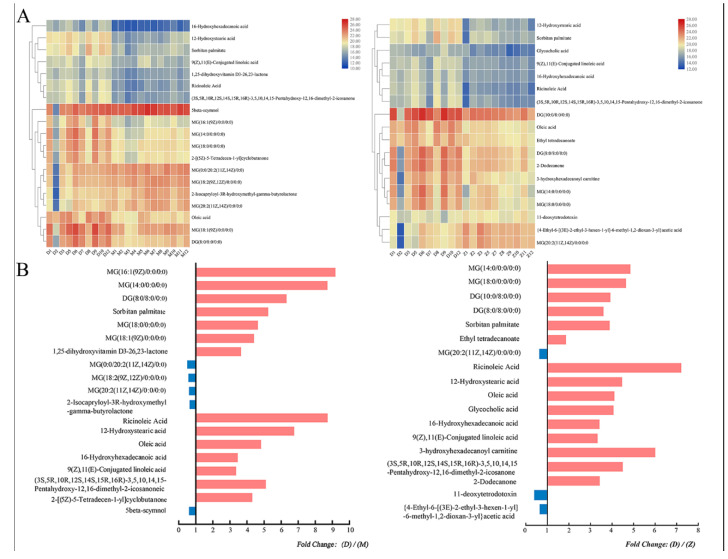
Hierarchical clustering analysis (**A**) and fold change (**B**) of the identified differential metabolites. Left: Significant different metabolites between group D (Mediterranean buffalo) and M (Murrah buffalo); right: Significant different metabolites between group D (Mediterranean buffalo) and Z (crossbred buffalo).

Metabolites with variable importance in the projection (VIP) values above 1, *p*-value < 0.05 and Fold change (FC) value > 2 or <0.5 were screened as significant different metabolites. Combining the ESI± LC-MS/MS data, 19 significant different metabolites were found between D and M, and 18 were found between D and Z. However, no significantly different metabolites were screened between M vs. Z (Supplementary Materials Tables S1 and S2). Among the differential metabolites, 11 were found to be both significantly different between D vs. M group and D vs. Z group (Supplementary Materials Figure S3), indicating that the 11 metabolites can be used as candidate biomarkers of Mediterranean buffalo milk. Further investigation indicated that most of the differential metabolites were glycerides or fatty acids. Moreover, unsupervised hierarchical clustering analysis also showed a clear separation of M, D, and Z buffaloes (Figure 3). The milk from D group had a higher level of MG (16:1(9Z)/0:0/0:0), ricinoleic acid and MG (14:0/0:0/0:0) compared to the Z group. While higher levels of ricinoleic acid, 3-hydroxyhexadecanoyl carnitine, and MG (14:0/0:0/0:0) in D group were found compared to the Z group (Figure 3). 

### 3.4. Integration of Key Different Metabolic Pathways 

**Figure 4:**
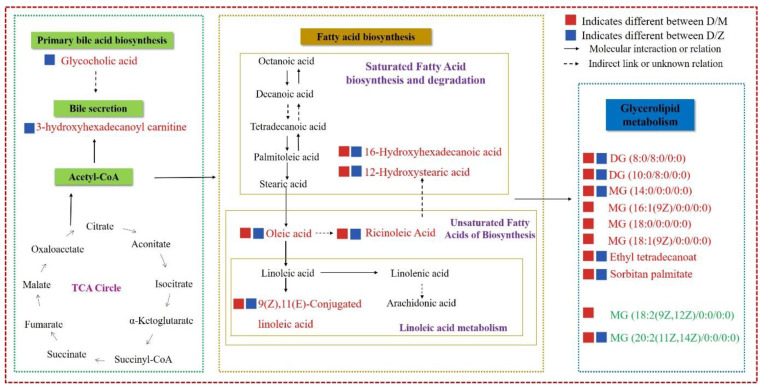
Metabolic network of the potential biomarkers which were different among group D (milk from Mediterranean buffalo), group M (milk from Murrah buffalo), and group Z (milk from crossbred buffalo). The increased metabolites in group D are labeled in red and the decreased metabolites are labeled in green.

In order to further investigate the function of the differential metabolites, MetaboAnalyst 4.0 was used to obtain the relevant pathways. Results showed that the differential metabolites were mainly involved in fatty acid metabolism pathways, such as fatty acid biosynthesis, unsaturated fatty acid biosynthesis, and linoleic acid metabolism (Table 2). In addition, differential metabolites were also enriched in the primary bile acid biosynthesis between D vs. Z group. A schematic diagram showing the metabolic network of the potential biomarkers which were different among the groups is shown by combining the identified differential metabolites and enriched pathways (Figure 4). 

## 4. Discussion

Buffalo milk is commercially more viable for producing high fat-based and solids non-fat based dairy products due to its high contents of fat, crude protein, and total solids [2]. Studies have found that milk traits are different among different buffalo species and can be affected by diverse factors [26,27,28]. Metabolomics has been used in many fields in recent years and reveals that the metabolome of buffalo milk may provide further understanding about the buffalo lactation mechanism. In this study, we analyzed the milk composition of Mediterranean, Murrah, and crossbred buffalo and found that the fatty acid in Mediterranean milk was significantly higher than Murrah buffalo and crossbred buffalo, while the protein and total solid content in Mediterranean milk were lower than the other groups. This result is consistent with the previous reports [29,30]. We further identified the metabolites in the milk by LC-MS/MS, and multiple strategies were used to analyze the data, including the construction of PCA and OPLS-DA models and permutation analysis. It was interesting to find that the differential metabolites between Mediterranean and Murrah or crossbred milk were mainly enriched in glycerides and fatty acids, such as oleic acid, 9(Z),11(E)-conjugated linoleic acid, MG (14:0/0:0/0:0), and MG(18:0/0:0/0:0), which are crucial in the process of milk fat synthesis. These differential metabolites are widely distributed in the milk fat synthesis pathways, including fatty acid biosynthesis, unsaturated fatty acid biosynthesis, and linoleic acid metabolism, indicating that the priority of different pathways affect the milk fat content in different buffalo species. Therefore, the higher abundance of lipid metabolism related metabolites in Mediterranean milk resulted in its higher fat content. No significant differences of milk content were found between Murrah and crossbred buffalo milk, as for the differential metabolites in this study. These results indicated that the hybrid buffalo inherited the milk-producing traits of Murrah buffalo, and also provide evidence on the effect of the hybrid buffalo in improving the milk-producing traits of local buffalo. 

At present, metabolomics has been applied to the screening of biomarkers and metabolic changes in various studies, such as heat stress in dairy cows [31] and pregnancy prediction of buffaloes [32], while few reports have focused on the metabolic differences between different buffalo species. In this study, we identified 19 and 18 significant different metabolites between the Mediterranean buffalo milk and Murrah or crossbred buffalo milk, respectively. Further analysis found that most of the screened differential metabolites were glycerides, and glyceride metabolism constitutes the final step of milk fat synthesis. The synthesis and secretion of milk fat is regulated by a complex network, and direct or indirect influence exists among the regulatory factors, while the specific mechanism still needs to be further explored [33]. The physiological phenomenon reflected by the change of metabolites is beneficial to the study of the mechanism of the biological process [34]. In mammary epithelial cells, glycerides are synthesized by combining the fatty acids and glycerol 3-phosphate under the sequential action of three enzyme families (glycerol-3-phosphate acyltransferases, GPATs; 1-acylglycerol-3-phosphate acyltransferases, AGPATs; and diacylglycerol acyltransferases, DGATs), and the accumulation of glycerides forms lipid droplets which finally secrete into the milk [35,36,37]. As an important precursor of milk fat synthesis, the difference in fatty acids lays the foundation for the difference of fat content among different buffalo species. Previous studies have found that the expression of SCD1 can directly or indirectly regulate the synthesis of fatty acid and triglyceride in the mammary epithelial cells by exploiting diverse metabolites [38,39]. Oleic acid (OA) is one of the principal UFAs in buffalo milk and an important substrate of enzymes such as diacylglycerol acyltransferase (DGAT) [40]. Astrocytes present a vital role in neural lipid metabolism for the regulation of energy balance to supply fatty acids and ketone bodies to other neural cells, and OA has been shown to be a potent inducer of astrocytic lipid droplet accumulation among various fatty acids [41]. OAs can promote morphology changes and lipid accumulation in the process of 3T3-L1 preadipocytes differentiation, and impact the methylation of transcription factors involved in lipid synthesis (Pparγ and C/ebpα) as well as motivate their expression in a dose-dependent manner [42]. 9(Z),11(E)-CLA is a major conjugate linoleic acid (CLA) and plays an important role in human health [43,44]. It can inhibit the transcription of pro-inflammatory cytokines by motivating the expression of Peroxisome proliferator-activated receptor gamma (PPARγ) and finally reduce the inflammation in mammary epithelial cells [45]. Studies have shown that UFAs can promote the deposition of TAG by regulating the fat synthesis related gene expression in mammary epithelial cells [37,46,47]. Comparing with Murrah buffalo milk, greater enrichment of MG (16:1(9Z)/0:0/0:0), ricinoleic acid, and MG (14:0/0:0/0:0) were found in Mediterranean milk in the present study. While a higher abundance of ricinoleic acid, 3-hydroxyhexadecanoyl carnitine, and MG (14:0/0:0/0:0) were found compared to the crossbred buffalo milk. Moreover, the higher abundance of glycerides is consistent with the result that Mediterranean milk contains more fat content in this study. Therefore, we can speculate that pathways such as fatty acid biosynthesis, unsaturated fatty acid biosynthesis, and linoleic acid metabolism in Mediterranean buffalo were more effective than in Murrah and crossbred buffalo.

## 5. Conclusions

Overall, we obtained the metabolite profiles of the milk from Mediterranean buffalo, Murrah buffalo, and crossbred buffalo (Murrah buffalo × local swamp buffalo) by UPLC-Q-Orbitrap HRMS. We identified 11 significantly different metabolites among the milks of the three buffalo species. These differential metabolites were found enriched mainly in the lipid synthesis pathway and may provide an explanation of the higher fat abundance in Mediterranean buffalo milk. The results provide further understanding of the mechanism of milk fat synthesis and may be used as candidate biomarkers in the breeding of high milk fat buffalo. Further experiments are underway. In general, our study provides practical information for the development of buffalo genetic improvement and the foundation for future research on specific milk nutrients.

## Figures and Tables

**Table 1:** Comparation of the main milk composition of Mediterranean, Murrah, and crossbred buffalo.

Breeds	Fat/%	Protein/%	Lactose/%	Total Solids/%
Mediterranean	8.40 ± 0.27 ^a^	4.32 ± 0.13 ^a^	5.12 ± 0.07 ^a^	19.05 ± 0.38 ^a^
Murrah	7.37 ± 0.32 ^b^	4.69 ± 0.12 ^ab^	5.13 ± 0.07 ^a^	17.26 ± 0.51 ^b^
Crossbred	6.87 ± 0.31 ^b^	4.82 ± 0.15 ^b^	5.24 ± 0.04 ^a^	17.44 ± 0.39 ^b^

Note: Different superscript letter indicates significantly different (*p* < 0.05).

**Table 2:** Metabolic pathways of differential metabolites of D vs. M.

Pathway Name	KEGG Map ID
Biosynthesis of unsaturated fatty acids	map01040
Fatty acid biosynthesis	map00061
Primary bile acid biosynthesis ∗	map00120
Linoleic acid metabolism	map00591

∗ indicates pathway only found in D vs. Z group.

## Supplementary Materials

The following are available online at https://www.mdpi.com/2076-2615/11/2/560/s1, Figure S1: Representative spectrum of milk samples from ultra-performance liquid chromatography quadrupole MS in ESI+ (A) and ESI− (B) mode. Figure S2: The correlation analysis of QCs in positive (A) and (B) negative ion mode. Figure S3: Venn analysis of the differential metabolites. Table S1: Significantly different metabolites between milk samples of Mediterranean buffalo and Murrah buffalo. Table S2: Significantly different metabolites between milk samples of Mediterranean buffalo and crossbred buffalo.
