# Supplementary material for: LC-MS/MS Based Metabolomics Reveal Candidate Biomarkers and Metabolic Changes in Different Buffalo Species
Source: Animals (Basel). 2021 Feb 20;11(2):560. doi: 10.3390/ani11020560 (PMC7924386; doi:10.3390/ani11020560)
Supplement: Supplementary file 1 [file animals-11-00560-s001.pdf]

Supplementary Material

LC-MS/MS based metabolomics reveal candidate biomarkers and Metabolic Changes in different buffalo species

Wen Shi, Xiang Yuan, Kuiqing Cui, Hui Li, Penghui Fu, Saif-Ur Rehman, Deshun Shi, Qingyou Liu, Zhipeng Li

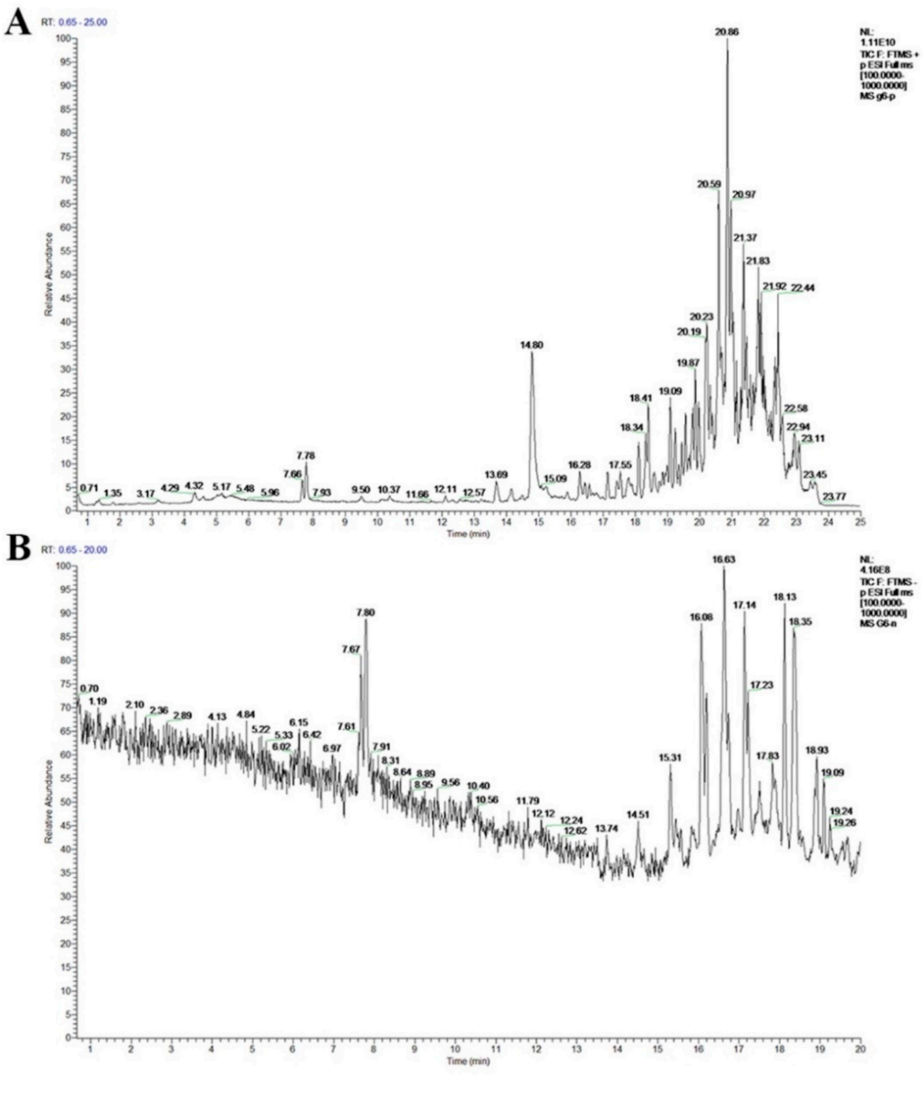

Figure S1. Representative spectrum of milk samples from ultra-performance liquid chromatography quadrupole MS in ESI+ (A) and ESI− (B) mode.

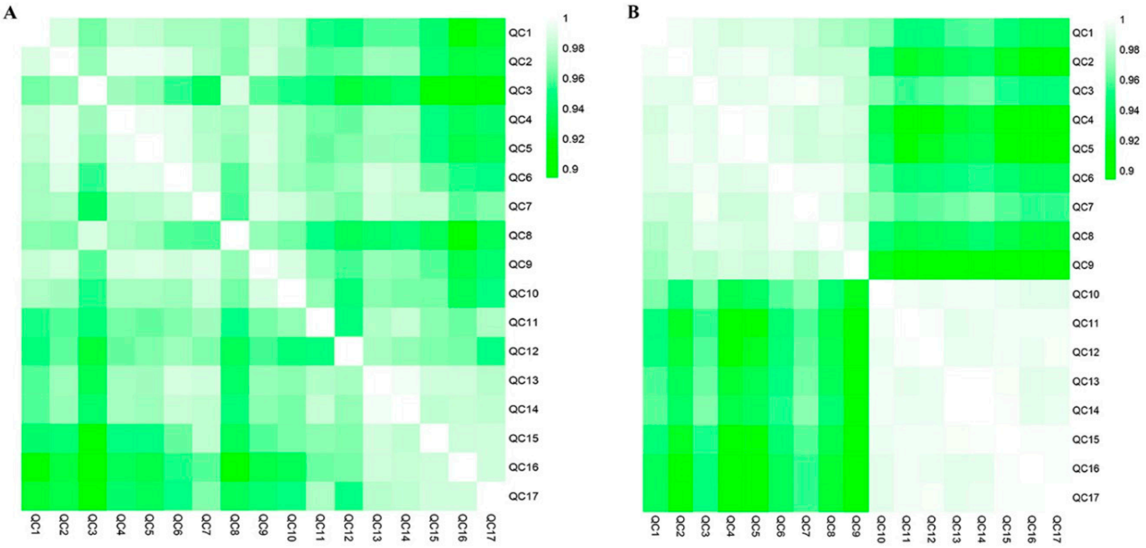

Figure S2. The correlation analysis of QCs in positive (A) and (B) negative ion mode.

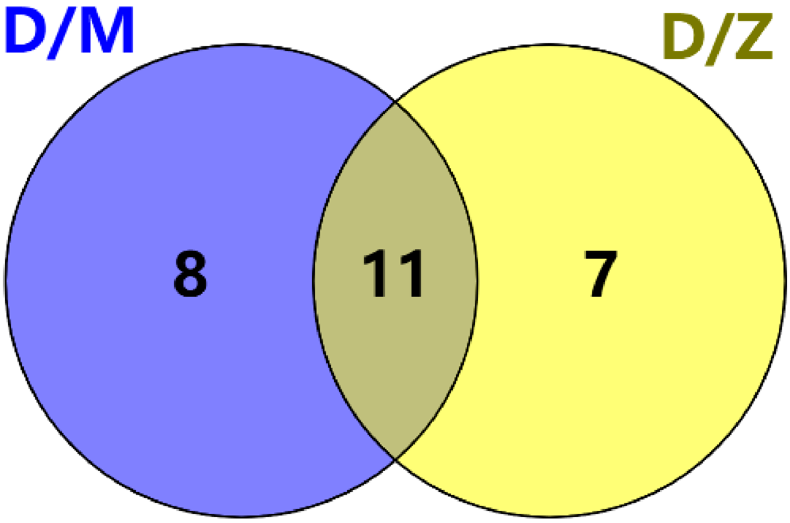

Figure S3. Venn analysis of the differential metabolites. D indicates milk samples from Mediterranean buffalo; M indicates milk samples from Murrah buffalo; Z indicates milk samples from crossbred buffalo.

Table S1. Significantly different metabolites between milk samples of Mediterranean buffalo and Murrah buffalo.

Compound Name	*p*-Value: (D) / (M)	VIP Value	Fold Change: (D) / (M)
Ricinoleic Acid	0.00005	1.752	8.736
16-Hydroxyhexadecanoic acid	0.00010	1.396	3.470
MG(16:1(9Z)/0:0/0:0)	0.00071	1.005	9.189
12-Hydroxystearic acid	0.00087	2.013	6.777
Sorbitan palmitate	0.00193	3.039	5.253
(3S,5R,10R,12S,14S,15R,16R)-3,5,10,14,15-Pentahydroxy-12,16-dimethyl-2-icosanone	0.00202	1.750	5.119
MG(18:0/0:0/0:0)	0.00409	1.268	4.646
MG(14:0/0:0/0:0)	0.00683	1.592	8.727
1,25-dihydroxyvitamin D3-26,23-lactone	0.00840	1.113	3.661

Oleic acid	0.00942	6.505	4.834
MG(20:2(11Z,14Z)/0:0/0:0)	0.01838	1.183	0.403
9(Z),11(E)-Conjugated linoleic acid	0.02619	1.169	3.378
MG(18:2(9Z,12Z)/0:0/0:0)	0.02647	1.567	0.428
5beta-scymnol	0.03184	4.562	0.407
MG(18:1(9Z)/0:0/0:0)	0.03672	2.739	4.429
DG(8:0/8:0/0:0)	0.03794	1.829	6.326
2-[(5Z)-5-Tetradecen-1-yl]cyclobutanone	0.03798	1.033	4.324
MG(0:0/20:2(11Z,14Z)/0:0)	0.04776	1.585	0.513
2-Isocapryloyl-3R-hydroxymethyl-gamma-butyro-lactone	0.04869	1.221	0.387

Table S2. Significantly different metabolites between milk samples of Mediterranean buffalo and crossbred buffalo.

Compound Name	*p*-Value: (D) / (Z)	VIP Value	Fold Change: (D) / (Z)
16-Hydroxyhexadecanoic acid	0.00009	1.369	3.430
Ricinoleic Acid	0.00010	1.711	7.227
Glycocholic acid	0.00108	1.095	4.085
12-Hydroxystearic acid	0.00126	1.950	4.483
9(Z),11(E)-Conjugated linoleic acid	0.00234	1.621	3.341
Oleic acid	0.00358	7.070	4.122
Sorbitan palmitate	0.00665	2.970	3.901
Ethyl tetradecanoate	0.00676	4.654	1.874
(3S,5R,10R,12S,14S,15R,16R)-3,5,10,14,15-Pentahy-droxy-12,16-dimethyl-2-icosanone	0.00719	1.705	4.509
MG(18:0/0:0/0:0)	0.00727	1.070	4.659
MG(14:0/0:0/0:0)	0.01705	1.357	4.863
2-Dodecanone	0.02009	1.617	3.444
DG(8:0/8:0/0:0)	0.02222	1.701	3.618
DG(10:0/8:0/0:0)	0.02396	3.570	3.939
11-deoxytetrodotoxin	0.04154	1.787	0.629
MG(20:2(11Z,14Z)/0:0/0:0)	0.04279	1.185	0.344
{4-Ethyl-6-[(3E)-2-ethyl-3-hexen-1-yl]-6-methyl-1,2-dioxan-3-yl}acetic acid	0.04462	1.214	0.353
3-hydroxyhexadecanoyl carnitine	0.04764	1.115	6.020
